# HapScoreDB: a database of protein language model functional scores for haplotype-resolved protein sequences

**DOI:** 10.1093/nar/gkaf1184

**Published:** 2025-11-20

**Authors:** Fabio Mazza, Filippo Gastaldello, Davide Dalfovo, Gianluca Lattanzi, Alessandro Romanel

**Affiliations:** Department of Cellular, Computational and Integrative Biology (CIBIO), University of Trento, Trento 38123, Italy; Department of Cellular, Computational and Integrative Biology (CIBIO), University of Trento, Trento 38123, Italy; Fondazione The Microsoft Research—University of Trento Centre for Computational and Systems Biology (COSBI), Rovereto 38068, Italy; Department of Cellular, Computational and Integrative Biology (CIBIO), University of Trento, Trento 38123, Italy; Department of Physics, University of Trento, Trento 38123, Italy; INFN-TIFPA, Trento Institute for Fundamental Physics and Applications, Trento 38123, Italy; Department of Cellular, Computational and Integrative Biology (CIBIO), University of Trento, Trento 38123, Italy

## Abstract

Deciphering the functional effects of genetic variants, especially those inherited together on the same haplotype, remains a major challenge in human genetics, where epistasis among co-occurring variants can further complicate interpretation. To address this, we present HapScoreDB, a database offering protein language model-derived scores for haplotype-resolved protein-coding sequences across all human transcript isoforms. Leveraging GENCODE and Ensembl annotations with phased variant data from the 1000 Genomes Project, HapScoreDB includes over 130 000 distinct protein haplotypes from >18 000 genes and 78 000 transcripts, encompassing over 94 000 coding variants. Fitness scores for each haplotype were computed using state-of-the-art protein language models. Preliminary analyses show that haplotypes harboring cancer GWAS variants tend to have significantly reduced predicted fitness. Moreover, variability in scores across haplotypes of the same transcript highlights known cancer genes, suggesting that dispersion in predicted fitness may capture functionally important variation. HapScoreDB features a user-friendly web interface for interactive exploration, visualization, and download of both full and customized datasets. As a dynamic and expandable platform, it connects real-world human genetic variation with advanced protein modeling, enabling novel approaches in variant interpretation, isoform prioritization, and population-scale functional genomics. Access HapScoreDB at https://bcglab.cibio.unitn.it/hapscoredb.

## Graphical Abstract

**Graphical Abstract:**
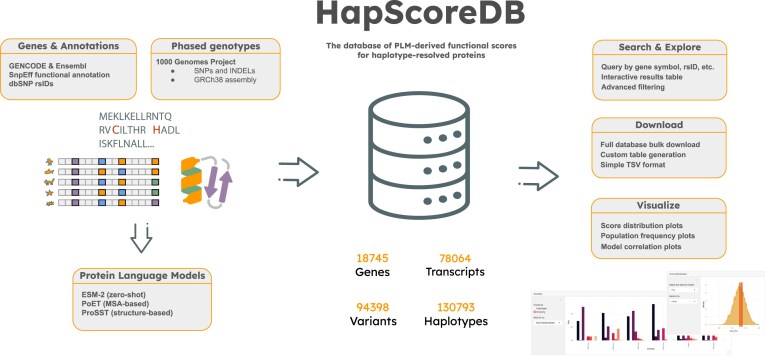

## Introduction

Protein-coding variation is a major contributor to diversity in the human proteome. Among the most frequent forms of genomic variations are single-nucleotide polymorphisms (SNPs) and small insertions and deletions (INDELs), which account for much of the phenotypic diversity observed among individuals. While the majority of variants are located in non-coding regions, where they can influence gene expression and regulation [1], those occurring in coding regions directly alter amino acid sequences, with potential consequences for protein structure, stability, molecular interactions, and function. Because of their direct impact on proteins, coding variants play a central role in human disease, including Mendelian disorders, complex traits, and cancer [2–5].

Historically, the functional effects of coding variants have been evaluated individually, focusing on single amino acid changes. Tools such as SIFT [6], PolyPhen-2 [7], and CADD [8] have provided valuable predictions of variant pathogenicity, yet they are inherently limited by their single-variant scope and cannot account for interactions between multiple variants within the same protein. In reality, coding variants frequently co-occur on the same haplotype and can interact within the same polypeptide chain. These intramolecular epistatic interactions can attenuate, amplify, or qualitatively alter the effect of individual mutations, shaping biochemical properties such as folding, enzymatic activity, and binding specificity [9–11]. Such interactions have important implications for evolution, disease risk, and therapeutic response [12–15], yet their combined functional impact remains challenging to predict.

Recent advances in deep learning have led to the emergence of protein language models (PLMs), which learn high-resolution representations of protein sequences through self-supervised training on large-scale sequence datasets [16]. Brandes *et al.* [17] first demonstrated the potential of PLMs, using ESM-1b [18] to predict single variant effects across the human proteome with results consistent with clinical data. Subsequent models such as MSA Transformer [19], PoET [20], SaProt [21], and ProSST [22] have improved predictive accuracy by integrating evolutionary and structural information. The ProteinGym benchmark [23] has facilitated systematic model evaluation, showing strong correlations between PLM predictions on deep mutational scanning (DMS) data and clinical phenotypes. It has also been observed that an increased performance of PLMs on DMS experiments, e.g. through the inclusion of evolutionary or structural data, results in better clinical classification as well [24]. A key strength of PLMs is their ability to process full-length protein sequences, enabling the assessment of multiple co-occurring variants within their complete sequence context.

At the same time, large-scale population sequencing projects such as the 1000 Genomes Project [25] and gnomAD [26] have produced extensive catalogs of human genetic variation, including phased genotypes. This has made it possible to reconstruct haplotype-resolved coding sequences and investigate protein diversity at the population scale. Notably, many naturally occurring protein haplotypes carry multiple amino acid substitutions, underscoring the need to move beyond single-variant analyses.

Here, we present HapScoreDB, a database integrating haplotype-resolved protein-coding sequences with functional predictions from state-of-the-art protein language models. By combining GENCODE [27] and Ensembl [28] transcript annotations with phased variant data from the 1000 Genomes Project, HapScoreDB reconstructs full-length protein sequences for over 130 000 distinct haplotypes spanning >18 000 human genes. Each haplotype is scored using multiple PLMs to estimate protein-level fitness, providing a population-scale view of the functional and evolutionary landscape of protein-coding variation.

By enabling systematic analysis of co-occurring coding variants in their haplotypic and protein context, HapScoreDB offers a new computational framework to investigate variant interactions, with applications in functional genomics, disease association studies, and precision medicine.

## Materials and methods

### Haplotype reconstruction and frequency calculation

Phased variant genotypes from the 1000 Genomes Project Phase 3 release (GRCh38/hg38) served as the primary data source [25]. For each protein-coding gene defined in GENCODE v47 [27], we extracted all genomic variants located within the exon regions of its associated transcripts. Variants were functionally annotated using SnpEff (v5.2f) [29] with the GRCh38 Ensembl 113 [28] database. We retained all variants predicted to alter the protein sequence, including missense, frameshift, stop-gain/loss, start-loss, and in-frame insertions/deletions, as well as synonymous variants. Variant identifiers (rsIDs) were assigned from dbSNP (build 156) [30] using SnpSift [31].

For each protein-coding transcript, haplotypes were reconstructed from the phased genotypes representing specific combinations of alternative alleles across all variant sites in the coding sequence (CDS). The wild-type haplotype was defined as containing no alternative alleles. Haplotype frequencies were calculated globally and for each of the five 1000 Genomes Project super-populations (AFR, AMR, EAS, EUR, SAS). Only haplotypes with a global frequency ≥0.5% were retained for scoring, with the wild-type haplotype for each transcript always included.

### Generation of haplotype-specific protein sequences

Haplotype-specific DNA sequences were generated by applying variants to the Ensembl reference transcript sequences. Nucleotide substitutions, insertions, and deletions were applied sequentially in 5′ to 3′ order. To ensure accuracy in the context of INDELs, a position shift tracker was implemented to maintain correct positioning for subsequent variants.

The resulting modified CDSs were translated into protein sequences using the standard genetic code. Special rules were applied for disruptive variants: for start-lost variants, the pipeline scanned for the next in-frame ATG codon to define the N-terminus; for premature stop codons, sequences were truncated; and for frameshift variants, translation continued in the new frame until the first subsequent stop codon. Each variant application was validated against the reference allele. Transcripts were excluded if they had incomplete CDS annotations, produced proteins shorter than 10 or longer than 4000 amino acids, or showed mismatches with the reference sequence. The upper length reflects performance constraints of the scoring pipeline and current PLMs.

### Functional scoring with protein language models

We employed three distinct PLMs to assign fitness-related scores to every generated protein sequence, selected to represent the three main categories of protein modeling: zero-shot (ESM-2 650M) [32], multiple sequence alignment (MSA)-based (PoET) [20], and structure-based (ProSST) [22]. These models were selected based on their state-of-the-art performance within their respective categories on established benchmarks such as ProteinGym [23].

PoET requires evolutionary context, so MSAs were generated for each wild-type sequence using MMseqs2 Release 17 [33] and the UniRef100 database [16], following the ColabFold [34] protocol, with a slightly lower sensitivity set to 7.5.

For ProSST, protein structures corresponding to the wild-type proteoforms were retrieved from the AlphaFoldDB [35], or otherwise predicted using Boltz 2.1 [36]. In the latter case, the same MSA built for PoET predictions was used as input, and three recycling steps were used to predict one diffusion sample per protein. Proteoforms corresponding to a Transcript Support Level of 5 and longer than 2000 amino acids were excluded. All the wild-type structures were then encoded into a sequence using the 4096-token-long ProSST structural encoder. The resulting structural sequences were used as inputs to all the respective haplotypes, with the exception of in-frame deletions and frameshifts, where we deleted from the input structural sequence the excess tokens in the respective positions. Insertions and other sequence modifications leading to an increase in the length of the sequence were left unmodeled.

For ESM-2 and ProSST, we computed the pseudo-log-likelihood (PLL) for each transcript-specific protein sequence *x* of length *L*, defined as the sum of the log-likelihoods of the amino acids in the sequence:


(1) \begin{eqnarray*}
\mathrm{ PLL}\left( x \right){\mathrm{ = }}\mathop \sum \limits_{i{\mathrm{ = }}1}^L \mathrm{ log}\left( {p{\mathrm{(}}{{x}_i}{\mathrm{|}}x)} \right).
\end{eqnarray*}

For PoET, an autoregressive model, we averaged the forward and backwards log-likelihoods, as well as likelihoods from MSAs of varying depth and diversity, following [20]. We refer to the resulting score associated with each sequence as PLL for simplicity.

The impact of a given mutated transcript-specific protein sequence (${{x}^{\mathrm{ mt}}}$) was then quantified by computing the difference between its *PLL* score and that of a reference sequence, denoted as pseudo-log-likelihood ratio (PLLR). We calculated two such metrics: $\mathrm{ PLLR}_{\mathrm{ wt}}$, where the reference is the wild-type transcript-specific protein sequence (${{x}^{\mathrm{ wt}}}$), resembling the score introduced in [17], and $\mathrm{ PLLR}_{\mathrm{ mf}}$, where the reference is the most frequent transcript’s haplotype in the human population (${{x}^{\mathrm{ mf}}}$):


(2) \begin{eqnarray*}
{\mathrm{ PLLR_{{wt}}}}\left( {\mathrm{ \mathit{ x}^{mt}}}\right) = \mathrm{ PLL}\left({\mathrm{ \mathit{ x}^{mt}}}\right)-\mathrm{ PLL}\left({\mathrm{ \mathit{ x}^{wt}}}\right),
\end{eqnarray*}

(3) \begin{eqnarray*}
{\mathrm{ PLLR_{{mf}}}}\left( {\mathrm{ \mathit{ x}^{mt}}}\right) = \mathrm{ PLL}\left({\mathrm{ \mathit{ x}^{mt}}}\right)-\mathrm{ PLL}\left({\mathrm{ \mathit{ x}^{mf}}}\right).
\end{eqnarray*}

These scores represent the predicted change in protein fitness, where lower values indicate greater predicted functional impairment. Additionally, to capture the overall functional variability of a transcript *t* across its allelic variants, we calculated a transcript-level $\mathrm{ PLL_{delta}}$ score. This score is defined as the difference between the maximum and minimum PLL scores observed across the set of all its constituent haplotypes (${{H}_t}$):


(4) \begin{eqnarray*}
\mathrm{ PLL_{delta}}\left( t \right){\mathrm{ = }}\mathop {\mathrm{ max}}\limits_{h\in {{H}_t}} \left( {\mathrm{ PLL}\left( h \right)} \right){\mathrm{ - }}\mathop {\mathrm{ min}}\limits_{h\in {{H}_t}} \left( {\mathrm{ PLL}\left( h \right)} \right).
\end{eqnarray*}

### AlphaMissense scores

To benchmark PLM predictions against a reliable measure of variant pathogenicity, we used AlphaMissense [37] substitution scores. Since the model weights are not publicly available, we relied on the released AlphaMissense database for human protein isoforms, extracting all entries corresponding to variants in haplotypes without INDELs. For each haplotype with matching AlphaMissense data, we obtained either a list of pathogenicity scores and classifications (for multiple missense variants) or a single score and classification (for haplotypes with one variant).

To derive a single haplotype-level pathogenicity score, we first reversed the logistic regression used to compute AlphaMissense probabilities, obtaining scores linearly related to the raw output logits:


(5) \begin{eqnarray*}
s = \textrm{logit}(\tilde{s}) = \mathrm{ ln}\left( {\frac{{\tilde{s}}}{{1 - \tilde{s}}}} \right).
\end{eqnarray*}

This transformation converts probabilities $\tilde{s}\ $to the log-odds scale, making them directly comparable to the pseudo log-likelihoods produced by the PLMs. For haplotypes with multiple missense variants, we then computed both the average and the sum of these logit scores to obtain a single representative value.

### Computational infrastructure

For the generation of haplotype-resolved protein sequences, PLM computations, MSA construction, and protein structure prediction, two distinct computational infrastructures were employed. The most computationally intensive tasks were executed on an HPE ProLiant DL380 Gen11 server, featuring dual Intel Xeon Gold 6530 processors, 512 GB of RAM, and two NVIDIA H100 GPUs with 94 GB of memory each. In addition, a high-performance workstation equipped with an Intel Core i9-10980XE processor and an NVIDIA RTX A5000 GPU with 24 GB of memory was used for complementary analyses.

### Analysis of cancer GWAS variants

Cancer GWAS coding variants were retrieved from the NHGRI-EBI GWAS Catalog [38] by filtering the “DISEASE/TRAIT” field for cancer-specific keywords. Each haplotype in HapScoreDB was then annotated as either “Cancer GWAS Variant” or “Non-Cancer GWAS” based on the presence of at least one rsID from the curated cancer-associated list. To robustly compare the functional impact scores between these two groups, we employed a bootstrap analysis of the median. For each group and different PLMs (ESM-2 and PoET), we generated 100 bootstrap replicates of the median $\mathrm{ PLLR_{wt}}$ score. The resulting distributions of these bootstrapped medians were visualized using boxplots combined with jitter plots to show both the central tendency and the sampling variability.

Furthermore, to explore the landscape of functional variability within the cancer-associated gene set, we analyzed the $\mathrm{ PLL_{delta}}$ scores. We generated a scatter plot to assess the correlation of $\mathrm{ PLL_{delta}}$ values between the ESM-2 and PoET models for all genes containing at least one cancer GWAS variant. Marginal density plots were added to visualize the distribution of $\mathrm{ PLL_{delta}}$ scores for each model independently.

All data processing and statistical analyses were conducted in R (v4.2.2) [39].

## Results

### Overview of HapScoreDB

**Figure 1.**
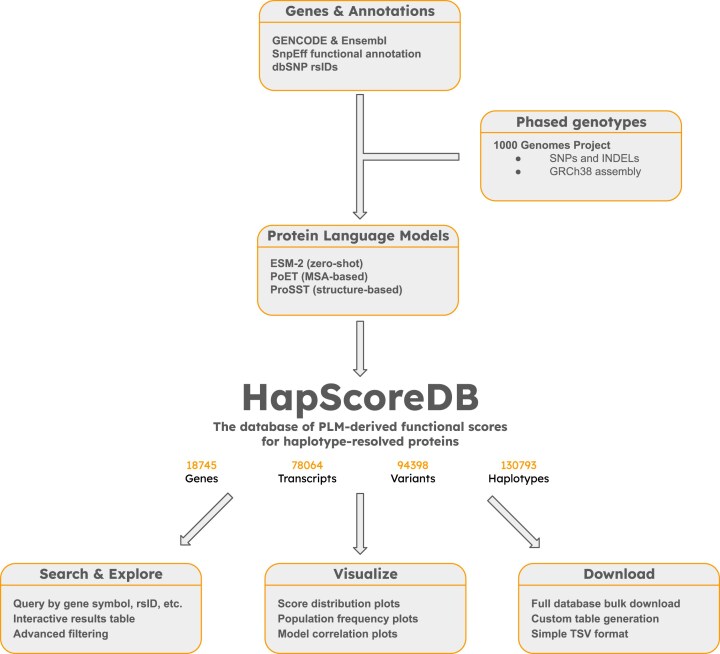
Overview of HapScoreDB database. The diagram illustrates the integration of input data sources, the core database elements, and the output resources available to the user.

HapScoreDB is a novel proteogenomic resource that integrates phased human genetic variation with functional predictions from state-of-the-art protein language models. The computational workflow used for database construction and querying is outlined in Fig. 1.

Starting from phased genotype data from the 1000 Genomes Project and transcript annotations from GENCODE and Ensembl, we reconstructed over 130 000 unique haplotype-resolved protein sequences. These sequences reflect the spectrum of protein diversity present in the human population. Each haplotype was evaluated using three representative PLMs (ESM-2, PoET, and ProSST) chosen to represent the major classes of current model architectures.

All resulting data, including functional fitness scores, population frequencies, and protein sequences, are compiled in HapScoreDB and made accessible via an interactive web portal designed to support data exploration, analysis, and download by the scientific community.

### Haplotype data characterization

HapScoreDB connects human genetic variation to predicted functional effects at the protein level by integrating haplotype-resolved coding sequences with deep learning-based scores computed using advanced protein language models.

The database contains a total of 359 697 entries, each representing a unique haplotype configuration observed across 78 064 protein-coding transcripts from 18 745 Ensembl genes, which correspond to 18 642 distinct HGNC gene symbols. These haplotypes collectively account for 130 793 non-redundant common combinations of coding variants and are annotated with 94 368 unique dbSNP rsIDs and 94 398 unique genomic variant coordinates based on the GRCh38 human reference assembly. Each entry links a transcript to a specific haplotype, comprising one or more nucleotide variants, allowing detailed representation of both single- and multi-variant configurations within protein-coding regions.

To ensure interpretability and cross-resource compatibility, all entries are annotated with Ensembl gene and transcript identifiers, as well as UniProt [40] protein accessions when available, covering ~80% of the dataset (41 813 unique UniProt IDs across ∼289 000 records). Each haplotype is associated with detailed molecular descriptors, including lists of reference and alternative alleles (8998 and 8768 distinct values, respectively), nucleotide-level changes (∼130 000 unique strings), and corresponding protein-level changes (∼147 000 unique consequences).

**Figure 2.**
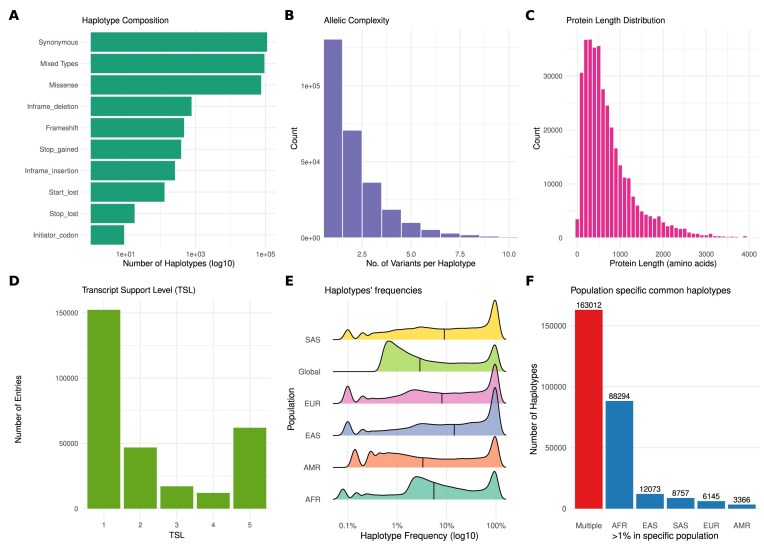
Characterization of the HapScoreDB haplotype data. (**A**) Haplotype composition based on the functional consequence of variants according to SnpEff annotations. The bar plot shows the count (on a log scale) of haplotypes for each category. (**B**) Distribution of allelic complexity, showing the number of variants contained within each haplotype. (**C**) Distribution of the length of protein sequences (in amino acids) present in the database. (**D**) Haplotype counts stratified by the transcript support level (TSL). (**E**) Distribution of haplotype frequencies (on a log scale) at a global level and within the five super-populations of the 1000 Genomes Project (AFR: African, AMR: Admixed American, EAS: East Asian, EUR: European, SAS: South Asian). (**F**) Number of haplotypes that are specific to a single population or shared across multiple populations.

We performed several analyses to characterize the database. An analysis of haplotype composition reveals the nature of variant combinations within the database (Fig. 2A). Most haplotypes consist of a single type of functional consequence, with synonymous-only (73 728) and missense-only (55 020) haplotypes being the most frequent. A considerable number, however, contain a mixture of different variant types, highlighting the importance of a haplotype-aware framework.

The allelic complexity, defined as the number of variants per haplotype, varies widely (Fig. 2B). While most haplotypes carry a single variant (∼130 000 entries), two-variant combinations are also common (70 866 entries), followed by a progressively decreasing number of higher-order combinations, up to a maximum of 68 variants per haplotype. This distribution underscores the combinatorial landscape of coding variation captured in the database.

The structural context of these variants is diverse, with protein lengths ranging from 10 to 3997 amino acids. The distribution is right-skewed, with a median length of 553 amino acids, indicating broad structural diversity (Fig. 2C).

To assess the reliability of the underlying transcript models, we incorporated the TSL from Ensembl. A large portion of the entries (152 282) are associated with the highest support level (TSL = 1), indicating high confidence in the transcript–haplotype associations for downstream functional or clinical follow-up (Fig. 2D).

A key feature of HapScoreDB is the integration of allele frequency data, both globally and across the five major super-populations of the 1000 Genomes Project: African (AFR), Admixed American (AMR), East Asian (EAS), European (EUR), and South Asian (SAS). The frequency distributions vary considerably across populations, as visualized in the ridgeline plot (Fig. 2E). The African population shows a broader distribution with a notable proportion of higher-frequency haplotypes (median frequency ∼4.5%), while the East Asian population is more concentrated at lower frequencies (median ∼1.2%). The global median frequency is ~2.8%, and notably, 25% of all haplotypes have a global frequency below 1%, indicating a substantial representation of rare and low-frequency coding haplotypes.

To further investigate population-specific variation, we identified haplotypes with a frequency ≥1% in a single population (Fig. 2F). As expected, the analysis reveals that the African population has the largest number of population-specific haplotypes, followed by the European and East Asian populations.

Collectively, HapScoreDB provides a detailed landscape of protein-coding haplotypes, capturing their composition, complexity, structural context, and population specificity.

### PLM-derived score characterization

To assess the functional predictions generated by PLMs, we analysed score distributions and model concordance across all variant-carrying haplotypes.

**Figure 3.**
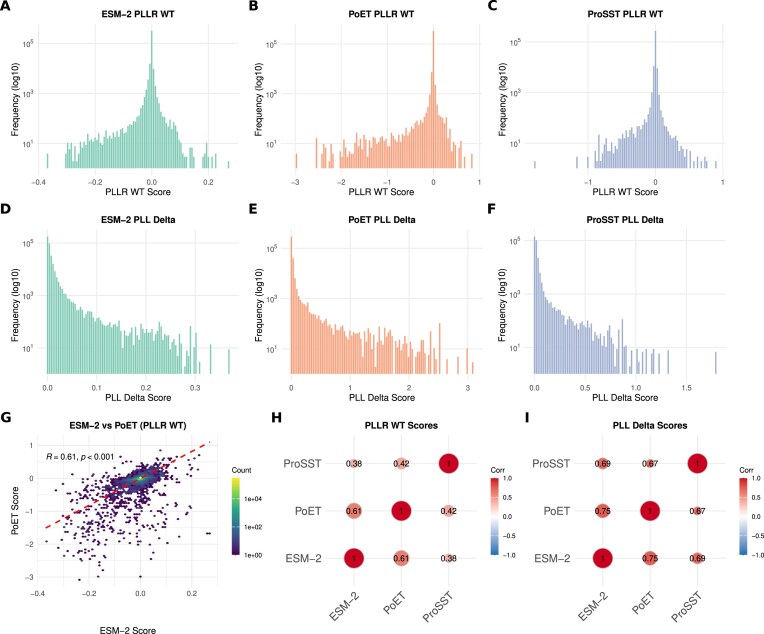
Distribution and correlation of PLM-derived functional scores. (**A**–**C**) Histograms showing the distribution of $\mathrm{ PLLR_{wt}}$ scores for the ESM-2, PoET, and ProSST models. (**D**–**F**) Histograms showing the distribution of $\mathrm{ PLL_{delta}}$ scores for the ESM-2, PoET, and ProSST models. **(G)** Scatter plot showing the correlation between the $\mathrm{ PLLR_{wt}}$ scores of ESM-2 and PoET. (**H, I**) Correlation matrices displaying the Pearson correlation coefficients between the $\mathrm{ PLLR_{wt}}$ and $\mathrm{ PLL_{delta}}$ scores for all model pairs.

First, we examined the distributions of the $\mathrm{ PLLR_{wt}}$ and $\mathrm{ PLLR_{mf}}$ scores for each model. As expected, these scores are largely centered around zero, indicating that a substantial fraction of haplotypes are predicted to be functionally neutral. However, the distributions exhibit long tails of negative scores, corresponding to haplotypes predicted to have a significant deleterious impact (Fig. 3A–C and Supplementary Fig. 1). We also examined the distributions of the $\mathrm{ PLL_{delta}}$ scores, highlighting the presence of a long tail of positive values indicating transcripts with high intra-variability (Fig. 3D–F).

A direct comparison between the $\mathrm{ PLLR}$ and scores of ESM-2 and PoET reveals a good linear relationship and high Pearson correlation (*R* > 0.6, *P*-value <.001), indicating that the models have a similar baseline assessment of protein sequence likelihood (Fig. 3G and Supplementary Fig. 2). Concordance persists across all three models (Fig. 3H and Supplementary Fig. 3), though ProSST scores show greater variability. Notably, the generally high correlation of $\mathrm{ PLL_{delta}}$ scores across all models (Fig. 3I) suggests that, despite their architectural differences, the models generally agree on the magnitude and direction of the functional impact.

To further evaluate the robustness of the observed concordance, we conducted stratified correlation analyses across different genetic contexts. First, we examined the correlation of PLLR scores as a function of allelic complexity (Supplementary Fig. 4), finding that the agreement between PLM models remains high for haplotypes carrying one, two, three, or more variants. We then stratified the analysis by variant type, focusing specifically on those that alter amino acid sequences (Supplementary Fig. 5). The correlation between PLLR scores remains robust for haplotypes with missense, stop-gained, frameshift, or mixed variant types. In contrast, this correlation moderately decreases for haplotypes composed exclusively of start-lost variants or INDELs. Notably, for uncommon categories like start-lost and in-frame insertion variants, the correlation improves upon excluding outliers, an effect likely attributable to the small number of observations (Supplementary Fig. 6). Finally, the models showed poor agreement for stop-lost and initiator-codon variants; however, the extremely limited number of observations (<20) for these categories precludes any definitive conclusions.

We then compared PLL scores with AlphaMissense pathogenicity predictions on haplotypes carrying only missense variants with available scores. AlphaMissense covers ∼40% of Ensembl transcripts and only 32% of HapScoreDB non-wild-type haplotypes. For overlapping cases, we averaged variant-level logits to obtain haplotype-level values. As expected, PLL scores showed negative correlations with AlphaMissense predictions, strongest with PoET (ρ = −0.60), weaker with ESM-2 (ρ = −0.35) and ProSST (ρ = −0.1). This underscores that different PLMs can provide partially orthogonal predictions that can reinforce each other or highlight different aspects of protein fitness, including potential epistatic interactions. The type of input the model receives is particularly important, and the fact that PoET scores are the most correlated with AlphaMissense predictions is consistent with the emphasis given to the MSA reconstruction objective used in the AlphaMissense pretraining.

Taken together, these findings support the robustness of PLM-derived scores in HapScoreDB and emphasize their value in offering complementary perspectives, particularly in contexts where predictions are less consistent.

### Web interface and usage

To facilitate data exploration and retrieval, we have developed a user-friendly web interface. The portal was developed in Shiny (v1.11.0) [41] using R (v4.2.2) and RStudio [42]. The interface is designed to provide powerful access to the database without requiring programming expertise and includes several pages for querying, visualization, and download, alongside comprehensive FAQ and contact pages.

**Figure 4.**
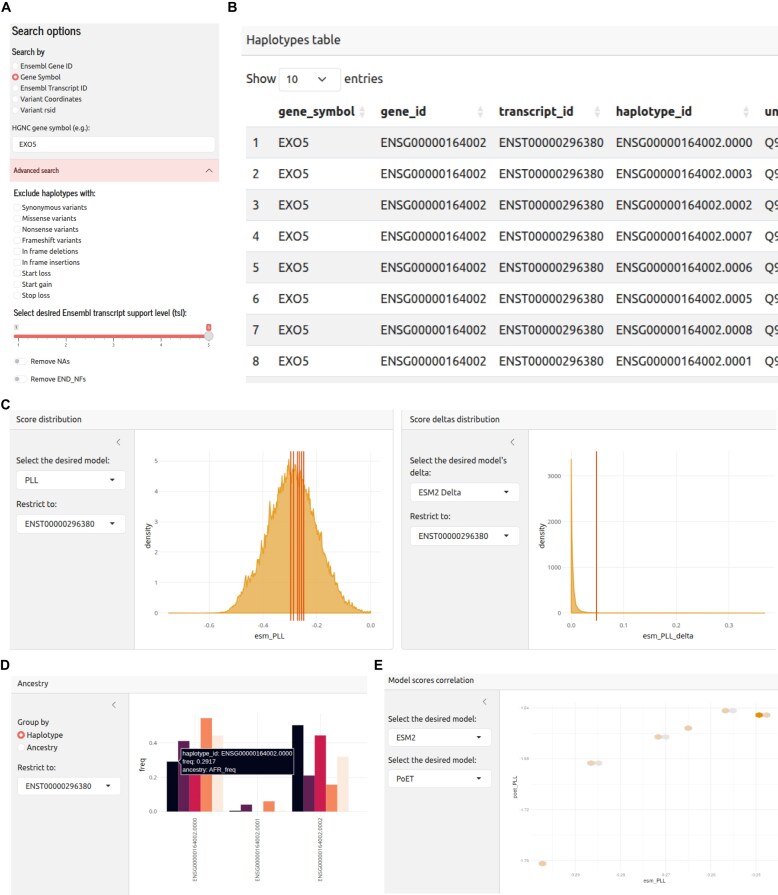
Overview of the HapScoreDB web portal interface and functionalities. (**A**) Main search panel, allowing users to query the database by gene (Ensembl ID or HGNC symbol), transcript ID, variant coordinates, or rsID and to perform advanced search options that allow for filtering results based on variant type (e.g. synonymous, missense) and TSL. (**B**) Interactive results table displaying the identified haplotypes for the queried gene, along with their respective IDs and metadata. (**C**–**E**) Examples of graphical visualizations available in the portal, including the score distribution and the model scores correlation.

The primary entry point is the “Search” page, which allows users to explore the scores of specific haplotypes. The query can be performed using a variety of common identifiers, including Ensembl gene or transcript IDs, HGNC gene symbols [43], dbSNP rsIDs, or variant coordinates (in the format CHR:POS.REF > ALT). The search bar features an auto-complete function to assist users in finding valid identifiers. A series of advanced filters (Fig. 4A) are also available to further subset the data based on the structural effects of the variants or the TSL.

Upon submission, the query returns a results page organized for clarity and rapid assessment. A series of value boxes at the top display summary counts of the retrieved haplotypes, affected transcripts, and the number of unique variants involved. The core of the page is an interactive and sortable data table (Fig. 4B) that presents all information for the selected haplotypes, where variants include direct links to ClinVar [44] and GWAS [38] databases, and proteins are cross-referenced with UniProt [40]. This design allows for the rapid identification and prioritization of potentially impactful haplotypes; for instance, a researcher can sort by the ESM-2 $\mathrm{ PLL_{delta}}$ score to immediately bring the most likely deleterious variants to the top for further inspection. A comprehensive description of all available data columns is provided in Supplementary Table 1.

For in-depth investigation, the results page also features a suite of interactive plots designed to provide deep contextualization of the data. The score distribution plot allows users to visualize the scores of selected haplotypes (e.g. $\mathrm{ PLL}$, $\mathrm{ PLLR_{wt}}$) against the background distribution of all scores in the database (Fig. 4C), making it easy to assess if a variant’s predicted impact is unusual. A similar plot is provided for $\mathrm{ PLL_{delta}}$ scores (Fig. 4C). Both visualizations are fully customizable, allowing the user to select the PLM of interest (ESM-2, PoET, or ProSST) and to focus the analysis on a single transcript or haplotype. Next, a dedicated visualization displays the haplotype frequencies for each transcript returned by the search (Fig. 4D). It shows the frequency for the global population alongside the five major super-populations (AMR, EUR, SAS, AFR, and EAS), with a selector that allows users to switch between different transcripts. Finally, to allow users to directly assess the consistency of predictions between models, two correlation scatter plots are included (Fig. 4E). These plots display the correlation between the same score type ($\mathrm{ PLL}$ and $\mathrm{ PLLR_{wt}}$, respectively) across user-selected pairs of models, providing a direct measure of their concordance.

### Data download and rest API

To enable large-scale computational research and ensure maximum utility for the bioinformatics community, all data are available through the “Download” page. We provide two main options for data retrieval. Users can perform a bulk download of the entire HapScoreDB dataset as a single, gzipped tab-separated value (TSV) file. Alternatively, for more targeted needs, users can use the interface to select specific columns of interest, including the full DNA and amino acid sequences for each haplotype, and download a custom-generated table. The use of a plain-text TSV format ensures broad compatibility with virtually all data analysis platforms, promoting transparency, reproducibility, and the development of novel analytical methods by the community. In addition, HapScoreDB provides a REST API system that allows users to retrieve information on single or multiple genes and variants, enabling seamless integration into automated bioinformatics pipelines.

### Case study

**Figure 5.**
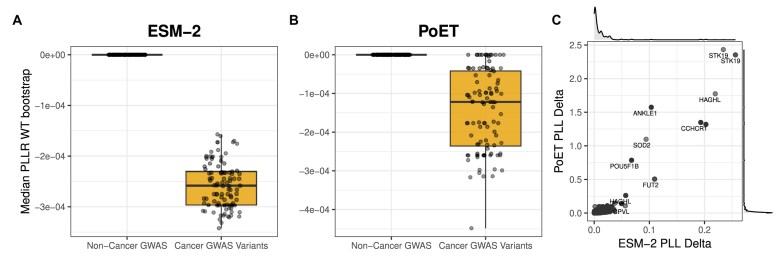
Functional analysis of cancer GWAS variants. (**A, B**) Boxplots comparing the distribution of functional fitness scores (median $\mathrm{ PLLR_{wt}}$ bootstrap) between haplotypes containing common non-cancer-associated variants (non-cancer GWAS) and haplotypes containing cancer risk variants (cancer GWAS variants) for the ESM-2 and PoET models. (**C**) Scatter plot comparing the $\mathrm{ PLL_{delta}}$ scores from ESM-2 and PoET for specific genes. Genes such as *FUT2, STK19, HAGHL*, and *ANKLE1* are highlighted, where GWAS variants lead to a predicted high $\mathrm{ PLL_{delta}}$ score by both models.

To illustrate the utility of HapScoreDB in dissecting the functional landscape of common variants associated with complex diseases, we performed a case study focused on haplotypes containing variants identified in cancer-related GWAS. Our first objective was to determine whether these common risk variants reside in haplotypes that exhibit distinct functional impact signatures. We conducted a bootstrap analysis of the median $\mathrm{ PLLR_{wt}}$ scores, comparing haplotypes containing at least one cancer GWAS variant to those without. The results show a clear and consistent trend across different PLMs. As shown in Fig. 5A and B, haplotypes carrying cancer GWAS variants show significantly lower median scores compared to haplotypes that do not carry cancer GWAS variants. This suggests that common variants associated with cancer risk, while not necessarily highly deleterious on their own, tend to be located within haplotypic contexts that are predicted to be more functionally impactful than the background.

We then analyzed the $\mathrm{ PLL_{delta}}$ scores, which capture the full dynamic range of functional impact across all haplotypes of a given gene transcript. Our PLM models consistently revealed a long tail of high $\mathrm{ PLL_{delta}}$ values (Fig. 5C), pointing to a subset of genes with exceptionally elevated scores. Notably, >10% of genes containing cancer-associated GWAS variants exhibit a $\mathrm{ PLL_{delta}}$ greater than 0.025, a threshold representing half of the $\mathrm{ PLL_{delta}}$ observed for *EXO5*, a gene in which we recently demonstrated that haplotypic variability has a strong functional impact on protein structure and dynamics [45]. One prominent example from this high $\mathrm{ PLL_{delta}}$ group is *FUT2*, a fucosyltransferase whose secretor or non-secretor status, determined by its activity, has been linked to susceptibility to various infections and is increasingly studied in the context of cancer risk [46, 47]. In our database, *FUT2* exemplifies this complexity, containing several distinct haplotypes formed by diverse combinations of cancer GWAS, non-cancer GWAS, and other coding variants. This suggests that different combinations might generate a wide spectrum of functional consequences on the resulting protein. Hence, while single risk alleles may confer a small increase in risk, specific combinations of these alleles within a single haplotype could lead to a much stronger deleterious effect, potentially creating a functional gradient of genetic predisposition to complex traits and diseases.

## Conclusion

In this work, we introduce HapScoreDB, a publicly accessible resource that provides functional fitness scores for over 130 000 non-redundant, haplotype-resolved protein sequences across the human genome. By leveraging multiple state-of-the-art protein language models, HapScoreDB advances functional annotation from a single-variant paradigm toward a more biologically relevant, haplotype-centric view [48]. In contrast to single-variant effect prediction tools, PLMs evaluate the entire protein sequence, allowing them to model the combined functional impact of co-inherited variants on a haplotype.

This shift enables a deeper understanding of how co-inherited genetic variants may act in concert to modulate protein function, addressing an essential, yet often overlooked, aspect of proteogenomic interpretations. HapScoreDB is designed as a flexible and extensible platform.

Our case study on cancer GWAS variants demonstrates the practical utility of HapScoreDB for hypothesis generation and variant prioritization. By analyzing haplotypes, our approach reveals how alleles with modest individual effects can combine to produce a significantly amplified functional impact, an effect often missed by single-variant annotations. Genes such as *FUT2* exemplify how complex combinations of common and rare variants can lead to a wide spectrum of predicted consequences.

Importantly, HapScoreDB introduces novel PLM-derived metrics, such as the $\mathrm{ PLL_{delta}}$ score, which quantifies the range of functional variability across transcript-associated haplotypes. This metric provides researchers with a powerful means to flag genes and regions where haplotype configuration is likely to influence protein function significantly.

Altogether, this haplotype-aware approach offers a new computational framework for investigating the local interaction of multiple germline variants.

While this novel framework is based on recent and powerful models, it is important to acknowledge the inherent limitations of the underlying PLMs. The accuracy of their predictions is often contingent on the availability of deep evolutionary data. Consequently, scores for orphan proteins or those with shallow MSAs may be less reliable. Furthermore, models that rely only on sequence information may not fully capture the effects of variants that cause subtle but critical disruptions to 3D structure, and those that do are dependent on the availability of reliable structures. However, HapScoreDB is designed as a flexible and extensible platform, and while it currently integrates three of the leading PLMs, its modular architecture supports future updates, including the integration of emerging models as they become available and validated. Some of the PLMs we plan to add are new versions of those already included (e.g. PoET2 [49]) and recent models that leverage multiple sources of information, such as MSA and structural data [50], or implement fine-tuning strategies based on complementary resources like deep mutational scanning [51]. This ensures continued relevance as both the modeling landscape and variant databases evolve.

By making these powerful but resource-intensive analyses broadly accessible, HapScoreDB can improve our understanding of the functional landscape of common genetic variants and their contribution to complex traits and diseases when integrated with orthogonal approaches [52, 53]. More broadly, it opens up new avenues for innovative strategies in variant effect prediction, isoform prioritization, and functional genomics at the population scale.

## Supplementary Material

gkaf1184_Supplemental_Files

## Data Availability

HapScoreDB is available at https://bcglab.cibio.unitn.it/hapscoredb. It requires no registration or login to access the data and use all of its features. Its content is updated in 6-month cycles for new models and major features and every 3 months for bug fixes and minor upgrades. Multiple sequence alignments, structures of processed proteoforms, and the code for the R Shiny application are publicly available on Zenodo at DOI 10.5281/zenodo.17358624 and at https://github.com/cibiobcg/HapScoreDB.
