# Supplementary material for: HapScoreDB: a database of protein language model functional scores for haplotype-resolved protein sequences
Source: Nucleic Acids Res. 2025 Nov 20;54(D1):D1087–97. doi: 10.1093/nar/gkaf1184 (PMC12807696; doi:10.1093/nar/gkaf1184)
Supplement: gkaf1184_Supplemental_Files [file gkaf1184_supplemental_files.zip › supplementary_figures.pdf]

Supplementary Figures

HapScoreDB: a database of protein language model functional scores for haplotype-resolved protein sequences

Fabio Mazza[1,†], Filippo Gastaldello[1,2,†] , Davide Dalfovo[1], Gianluca Lattanzi[3,4] and Alessandro Romanel[1,*]

[1] Department of Cellular, Computational and Integrative Biology (CIBIO), University of Trento, Trento, 38123, Italy
[2] Fondazione The Microsoft Research - University of Trento Centre for Computational and Systems Biology (COSBI), Rovereto, 38068, Italy
[3] Department of Physics, University of Trento, Trento, 38123, Italy
[4] INFN-TIFPA, Trento Institute for Fundamental Physics and Applications, Trento, 38123, Italy

† Joint Authors
* To whom correspondence should be addressed. Tel: 00390461285217; Email: alessandro.romanel@unitn.it

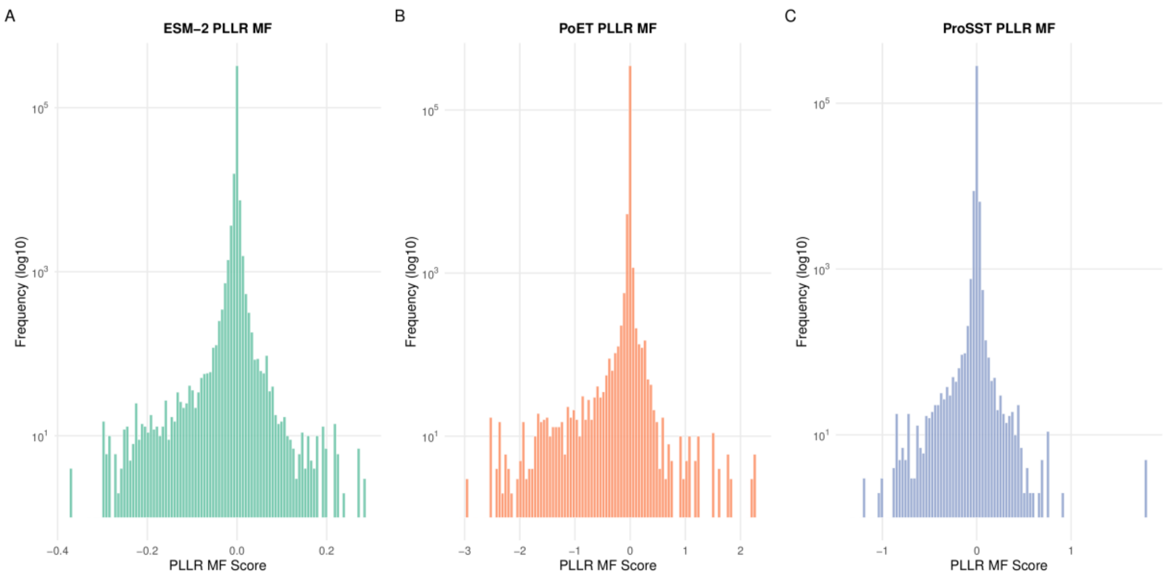

Figure S1: Distribution of functional fitness scores (PLLR). Histograms showing the distribution of $PLLR_{mf}$ scores for each of the three PLM used: **(A)** ESM-2, **(B)** PoET, and **(C)** ProSST. The y-axis represents the frequency on a logarithmic scale.

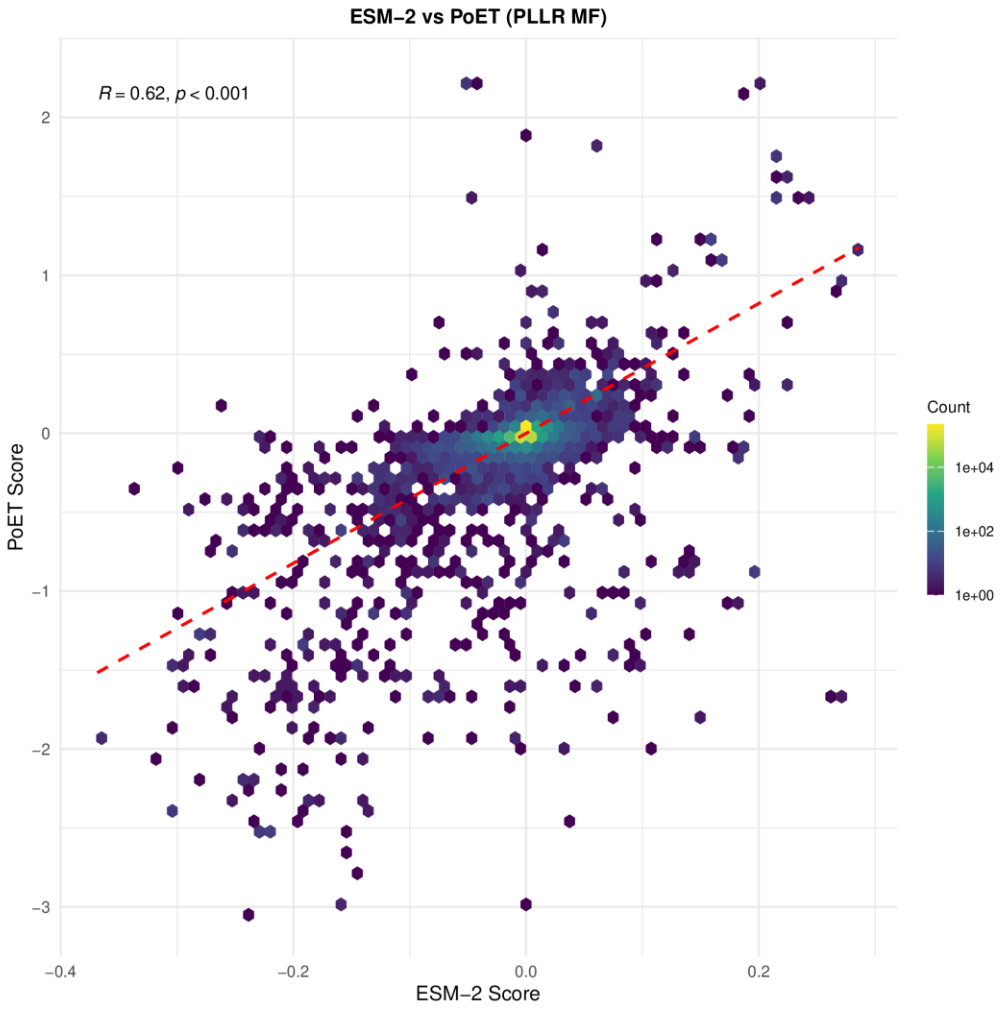

Figure S2: Correlation of fitness scores between ESM-2 and PoET. Scatter plot illustrating the correlation between $PLLR_{mf}$ scores predicted by the ESM-2 (x-axis) and PoET (y-axis) models. Each point represents a single haplotype.

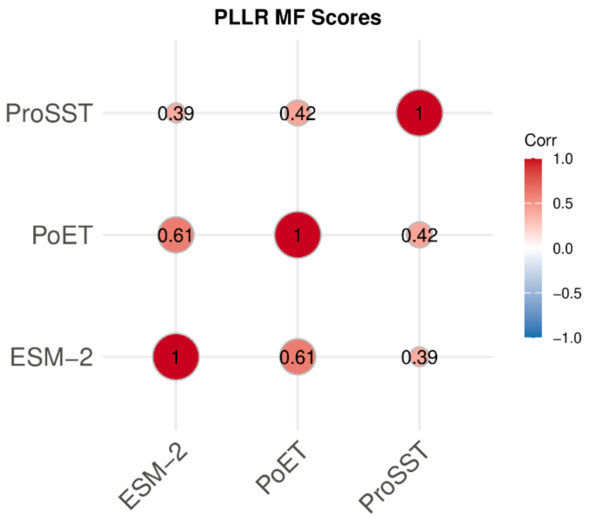

Figure S3: Correlation matrix of fitness scores across all models. Correlation matrix showing the Pearson correlation coefficients $PLLR_{mf}$ for all pairs of the three models: ESM-2, PoET, and ProSST.

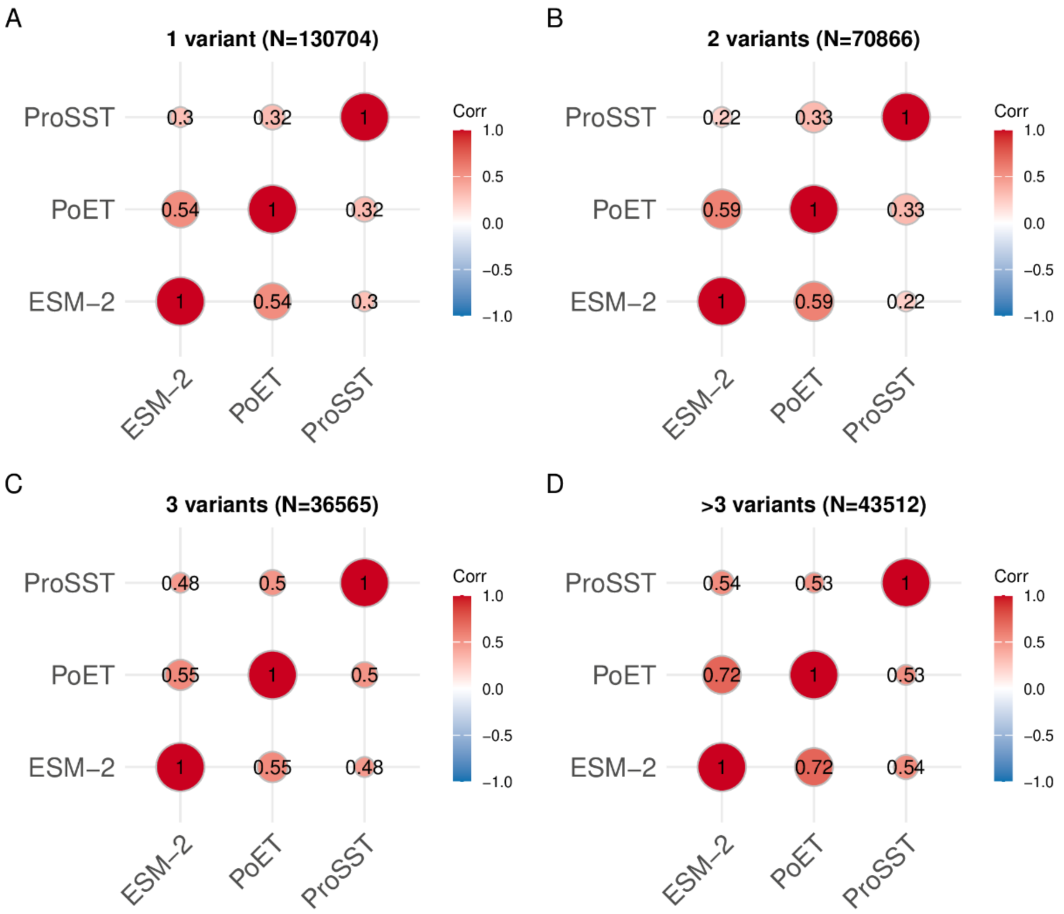

Figure S4: Correlation of scores stratified by allelic complexity. Correlation matrices of the functional fitness scores among the three $PLLR_{wt}$, stratified by the number of variants present in each haplotype. The panels show the correlation for haplotypes containing **(A)** 1 variant, **(B)** 2 variants, **(C)** 3 variants, and **(D)** more than 3 variants. Results for $PLLR_{mf}$ are identical.

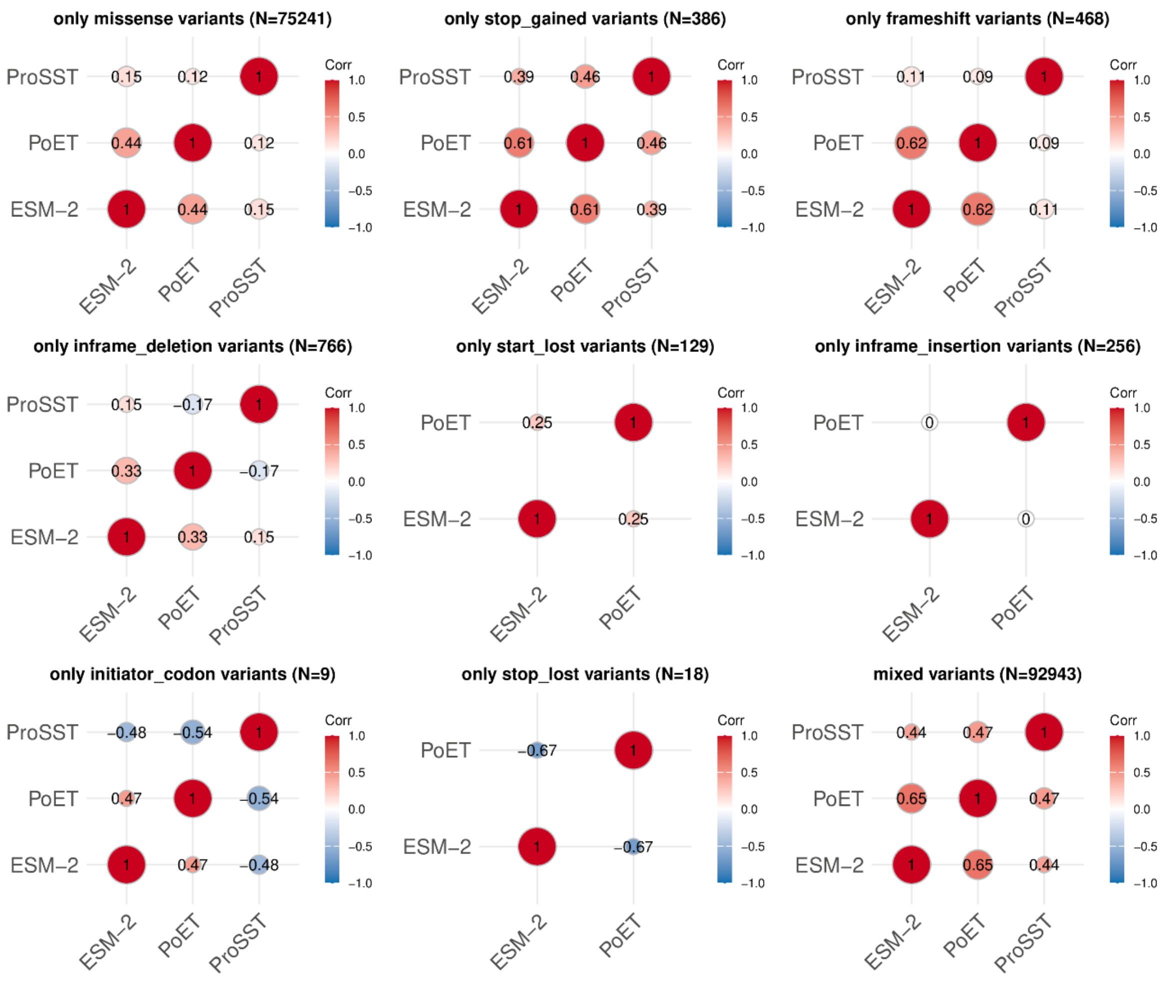

Figure S5: Correlation of scores stratified by variant functional consequence. Correlation matrices of the functional fitness scores among the three PLMs, stratified by the functional consequence predicted by SnpEff for the variants within each haplotype.

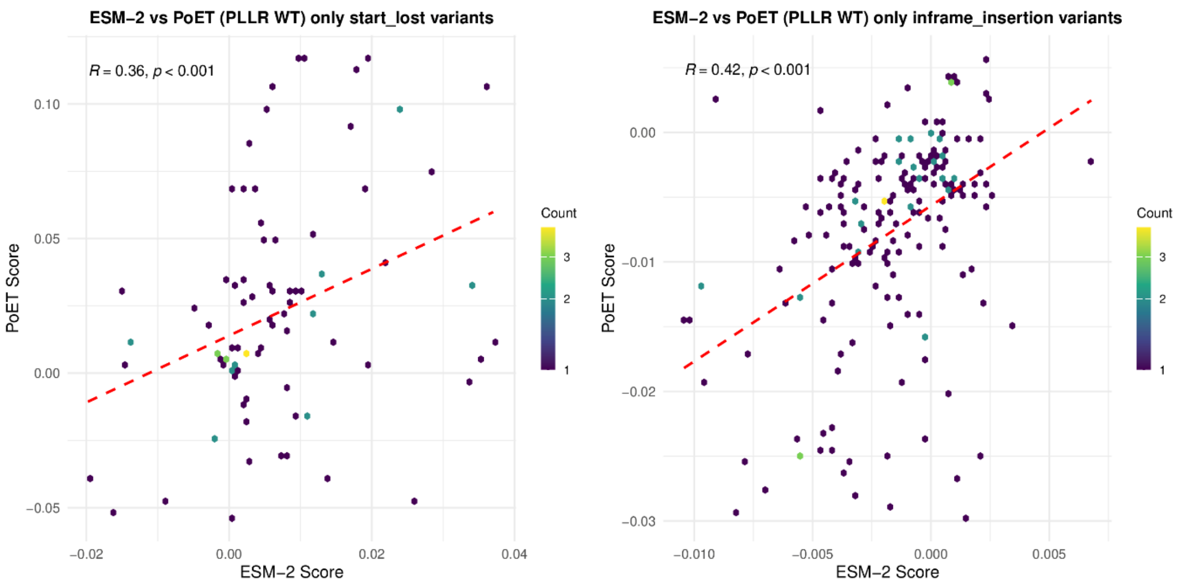

Figure S6: Correlation of fitness scores between ESM-2 and PoET for start_lost and inframe_insertion variants. Scatter plot illustrating the correlation between $PLLR_{wt}$ scores predicted by the ESM-2 (x-axis) and PoET (y-axis) models for start_lost and inframe_insertion variants. Each point represents a single haplotype.
